# Single-row versus transosseous technique in the arthroscopic treatment of rotator cuff tears: a meta-analysis

**DOI:** 10.1007/s00590-023-03657-0

**Published:** 2023-08-10

**Authors:** S. De Giorgi, G. Ottaviani, F. P. Bianchi, M. Delmedico, M. Suma, B. Moretti

**Affiliations:** 1 Department of Translational Biomedicine and Neuroscience “DiBraiN”, School of Medicine, University of Bari “Aldo Moro”, AOU Consorziale Policlinico, Piazza Giulio Cesare 11, 70124 Bari, Italy; 2 Interdisciplinary Department of Medicine, University of Bari, Bari, Italy

**Keywords:** Transosseous, Rotator cuff, Suture-anchor, Shoulder

## Abstract

**Purpose:**

This study aims to compare single-row suture-anchors (SA) versus transosseous arthroscopic (TO) technique in the treatment of patients with rotator cuff tears in terms of clinical structural outcomes at atleast 24 months of follow-up.

**Methods:**

The systematic review was performed according to “PRISMA guidelines” (Preferred Reporting Items for Systematic Reviews and Meta-analyses), in order to identify all the studies comparing clinical, both subjective and objective, outcomes with 24 months follow-up minimum in patients undergoing arthroscopic RC repair with the SR and TO technique. OVID-MEDLINE®, Cochrane, SCOPUS and PubMed were searched from January 2010 to October 2022 to identify relevant studies, using the following key words, that were combined together to achieve maximum search strategy sensitivity: “Rotator cuff tear” OR “repair” OR “shoulder” OR “reconstruction” OR “suture” OR “arthroscopic” OR “single-row” OR “transosseous”.

**Results:**

Six papers were finally analyzed in this meta-analysis. The weighted mean difference on Constant scores and for ASES for studies considering suture-anchors (SA) group showed good outcomes. The weighted mean difference of Constant scores and of ASES for TO (transosseous) group showed good outcomes. The weighted mean difference of CONSTANT for TO versus SA groups showed no differences in the outcomes of SA and TO techniques for the repair of Rotator Cuff Tears at minimum 24 months follow-up.

**Conclusions:**

The Arthroscopic transosseous rotator cuff repair technique and SA (suture-anchor) technique both lead to significant short-term improvement and satisfactory subjective outcome scores with low complication/failure rates. No differences were found in the final outcome between the two techniques.

## Introduction

Rotator cuff tears (RCT) are a common cause of pain of the shoulder and progressive functional limitation in the activities of daily life (ADL) especially in adult population. Only in the USA, more than 4 million patients per year require an orthopaedic examination because of shoulder pain [24]. Its incidence increases with aging, with an estimation of 30% of the population over 60 years suffering from it [17, 19, 21, 23, 35, 36].

Nevertheless, more than the 65% of the procedures are performed in patients with less than 65 years old [7, 10, 24, 27, 29, 32], with necessarily higher socio-economic and public healthcare costs [16, 20]. The rate of surgical treatments for RCT is furthermore growing, as demonstrated by an increase of 141% rotator cuff repairs from 1996 to 2006 only in the US [7]. In Italy approximatively 62 procedures every 100.000 inhabitants are performed [25, 33].

Open repair techniques, such as transosseous fixation that led the way, have been progressively replaced as the gold standard treatment for RCT with the advent of arthroscopy. Simultaneously, the number of arthroscopic techniques has spread [4, 5, 13, 34].

Suture anchors have been widely and safely used over the years thanks to their capacity to guarantee the rotator cuff tendons to the humeral footprint and has become, nowadays, the first choice in the treatment of arthroscopic rotator cuff repair.

Progressively, different materials and configurations have been tried—single-row (SR), double-row (DR) and to double-row transosseous equivalent (TOE), but none of them proved itself better than the others, as far as functional outcomes concerns, and in these days and age there is no guideline that indicates which one to use so the choice is often up to the surgeon confidence with a technique rather than the other [9]. The widely accepted concept of using more than one anchor in order to obtain a better repair has, perhaps, the inconvenience of increasing surgical time and implant costs.

SR anchor suture repair guarantees, however, the same functional and biomechanicals outcomes with reduced costs and surgical time compared to DR and TOE, reason why is a more diffusely applied procedure [1].

Problems observed with these suture-anchor techniques are: difficulties in case of revision surgery due to the presence of anchors in the greater tuberosity, short-term retear, anchor displacement, knot impingement and, even if less frequently, greater tuberosity bone osteolysis [1, 3, 18].

In the attempt to overcome these limitations, the transosseous (TO) open RCT repair originally described by McLaughlin in 1944, the principal procedure performed for decades, has been re-edited in an all-arthroscopic TO rotator cuff repair version with the aim to combine the biomechanical advantages of the open fixation method with the pros of a closed surgery [5, 11, 26, 30]. Another edge of this technique can be the possible release of stem cells and growth factors from the bone tunnel that can improve tendon healing [8].

The single-row suture-anchor repair consists in one or more anchors implanted onto the greater tuberosity of the humerus, containing two or three sutures passing through the tendon.

The Transosseous arthroscopic technique consists in one or more bone tunnels into the greater tuberosity of the humerus with generally three sutures passing through the tendon.

Even though in the literature only few studies analyze the outcomes arthroscopic TO rotator cuff repair, newly published reports have shown promising results, similar to those with anchor repair [2, 18].

Regardless various systematic reviews and meta-analyses during the years have been published studying the effectiveness of the conservative and surgical treatment, only few trials comparing the suture-anchor and TO techniques have been produced.

The purpose of the present study was to compare the clinical and functional outcomes of arthroscopic SR suture anchor and TO repair in the treatment of RCT in order to evaluate the possible superiority of one technique rather than the other.

We report a Meta-analysis about the transosseous rotator cuff repair technique and single-row with suture-anchors technique, to consider the best technique of rotator cuff tear repair.

## Methods

### Meta-analysis

The Meta-analysis was performed according to “PRISMA guidelines” (Preferred Reporting Items for Systematic Reviews and Meta-analyses), in order to identify all the studies evaluating clinical, both subjective or objective, outcomes with 24 months follow-up minimum in patients undergoing arthroscopic RC repair with the SR and/or TO technique [28].

### Search strategy

OVID-MEDLINE®, Cochrane, SCOPUS and PubMed were searched from January 2010 to October 2022 to identify relevant studies, using the following key words, that were combined together to achieve maximum search strategy sensitivity: “Rotator cuff tear” OR “repair” OR “shoulder” OR “reconstruction” OR “suture” OR “arthroscopic” OR “single-row” OR “transosseous”. A manual search of the reference lists of the selected publications was also performed, to identify additional studies for potential inclusion. Two reviewers (DM and OG) independently screened the titles, abstracts and the full texts for the inclusion of the studies in this review. Potentially relevant articles were acquired for full-length text and Authors were contacted when the article was not available.

### Eligibility criteria

Full-text articles alone published between January 2010 and October 2022 were included. The review was restricted to articles published in English. Inclusion criteria were: (1) human studies, (2) studies evaluating and/or comparing the SR and TO technique for the repair of RCT, (3) all levels of evidence, (4) studies with detailed clinical outcome, (5) at least 24 months follow-up. Exclusion criteria, on the other hand, were: (1) less or median 24 months of follow-up, (2) cadaveric/animal/in vitro/in vivo studies, (3) studies without clinical outcome, (4) other systematic reviews, (5) outcomes not indicated as average.

When multiple reports from the same center or trial were found, the most detailed publication was selected.

The PRISMA flowchart is presented in Table 1.

**Table 1:**
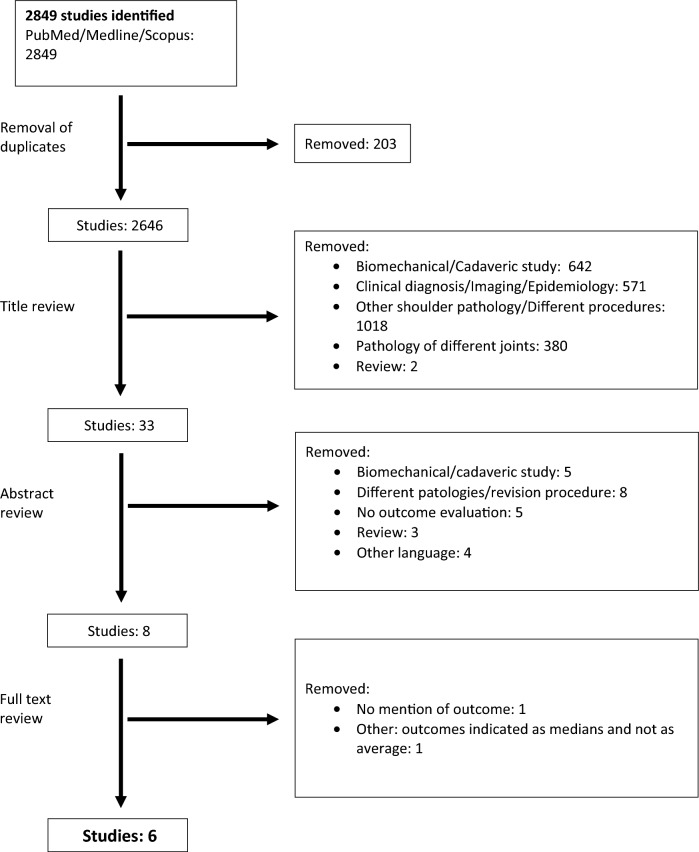
Flow chart

### Data extraction

Information were extracted from each study by two reviewing Authors (OG and SM) and collect in a Microsoft Excel sheet, then checked by two different Authors (DM and SM), including: (1) year of publication, (2) study design, (3) level of evidence, (4) number of patients, (5) characteristics of study participants (age, gender, BMI) and the study inclusion and exclusion criteria, (6) surgical technique, (7) clinical outcome (i.e. Visual Analog Scale, American Shoulder and Elbow Surgeon Score, Quick Disabilities of the Arm, Shoulder and Hand questionnaire, Constant–Murley Score), (8) radiological outcome, (9) postoperative complications. Disagreements between the reviewers were thoroughly examined; discrepancies were resolved by consensus discussion and mediation of the senior review Author (DGS), where needed.

### Inter-observer agreement assessment

To determine inter-reviewer agreement, Cohen’s Kappa (*K*) score was calculated after each screening stage. *K* score between 0.01 and 0.20 indicates slight agreement; a *K* score between 0.21 and 0.40 indicates fair agreement; *K* score between 0.41 and 0.60 indicates moderate agreement; *K* score between 0.61 and 0.80 indicates substantial agreement and *K* score between 0.81 and 0.99 indicates almost perfect agreement.

### Statistical analysis

The measure of the treatment effect is the changes in CONSTANT and ASES between baseline and endpoint values of the studies. When the SD value was not reported at baseline or endpoint, it was estimated as mean value of SD values reported in the other included studies. Where the mean and SD of the change from baseline to endpoint were not reported in the original articles, the following equations were used to calculate them.

$${\text{Treatment}}\;{\text{Effect}} = \overline{{X_{2} }} - \overline{{X_{1} }} ,$$

$${\text{SD}}_{{{\text{treatment}}\;{\text{effect}}}} = \sqrt {{\text{SD}}_{1}^{2} + {\text{SD}}_{2}^{2} - \left( {2 \times r \times {\text{SD}}_{1} \times {\text{SD}}_{2} } \right)}$$

where *r* represents the correlation coefficient. We took *r* = 0.4 as a conservative estimate in this study. The weighted mean differences (WMDs) with 95% CI were calculated for the continuous outcomes for each study. Because each outcome of interest was assessed separately, and the unit of measurement was the same across studies for the specified outcomes, the mean difference was not standardized.

An inverse-variance random-effects model was used. Forest plots were used to determine if there was variable specific efficacy heterogeneity. The *I*^2^ test was used to assess heterogeneity based on the thresholds reported in the Cochrane Handbook for Systematic Reviews of Interventions: 0–40% might not be important, 30–60% may represent moderate heterogeneity, 50–90% may represent substantial heterogeneity, and 75–100% may represent considerable heterogeneity. *P* value < 0.05 was considered statistically significant for heterogeneity. It was not possible to assess potential publication bias because of the few included studies; Furthermore, any sub-analysis for the quality was assessed for the same reason.

### Identification of relevant studies

We included 3 papers about the SA technique and another paper regarding TO technique [14, 15, 22, 34].

In the present Meta-analysis other two papers were collected comparing both the SA (Suture-anchors) technique and the TO (Transosseous) technique [6, 12]. Thus a total of 6 papers were analyzed.

The Levels of evidence for the 6 papers are showed in Table 2.

**Table 2:** Level of evidence of the included studies

References	Study design	Level of evidence
Castagna et al. [6]	Prospective	3
Garofalo et al. [12]	Retrospective	2
Iman [14]	Prospective	2
Jeong et al. [15]	Retrospective	3
Liu et al. [22]	Retrospective	3
Tashjian et al. [34]	Retrospective	3

The paper by Randelli was not included in our Meta-analysis because outcomes were indicated as medians and not average [31].

## Results

**Fig. 1:**
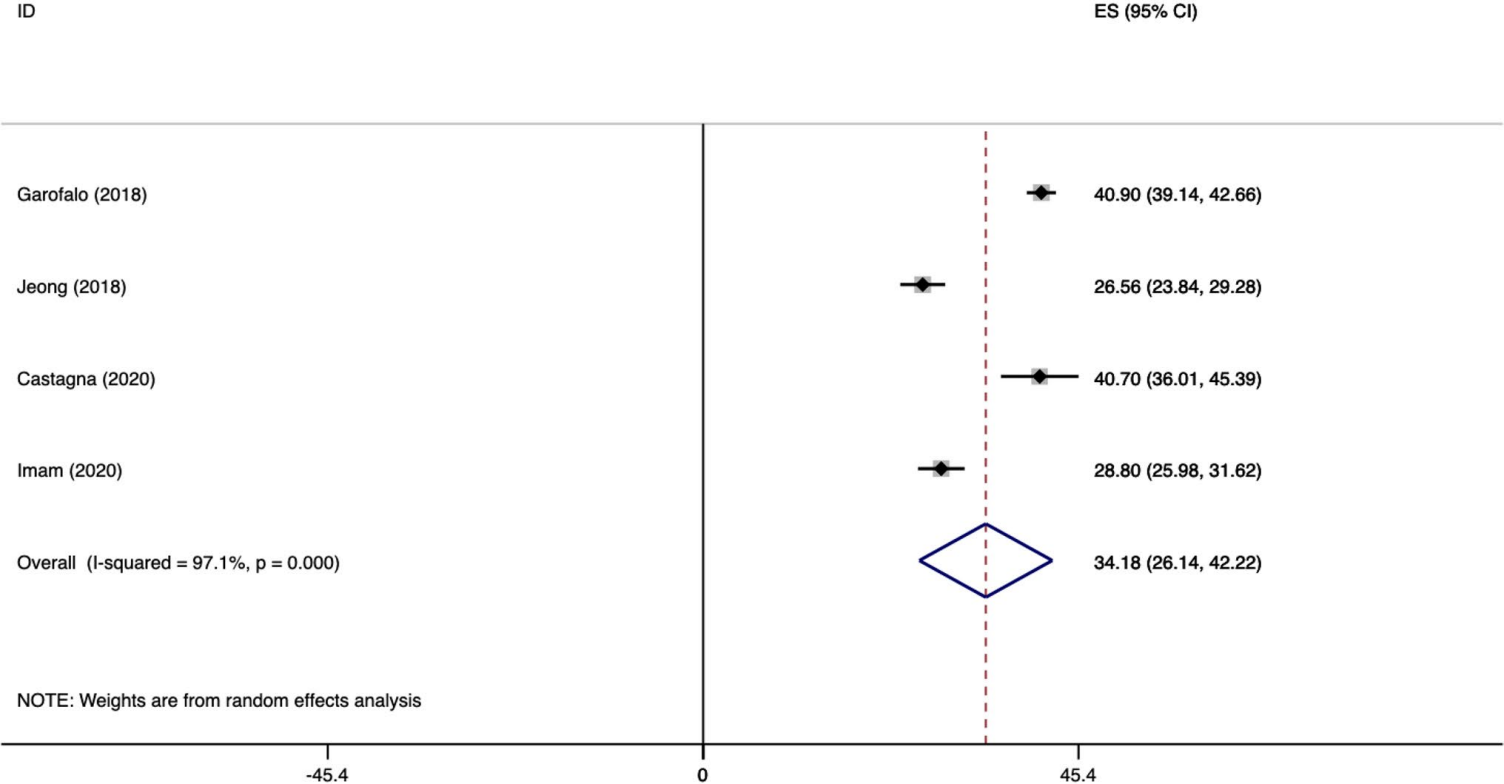
Weighted mean difference of CONSTANT score for SR group

The weighted mean difference on Constant scores for studies considering SA (suture-anchors) group is showed in Fig. 1.

**Fig. 2:**
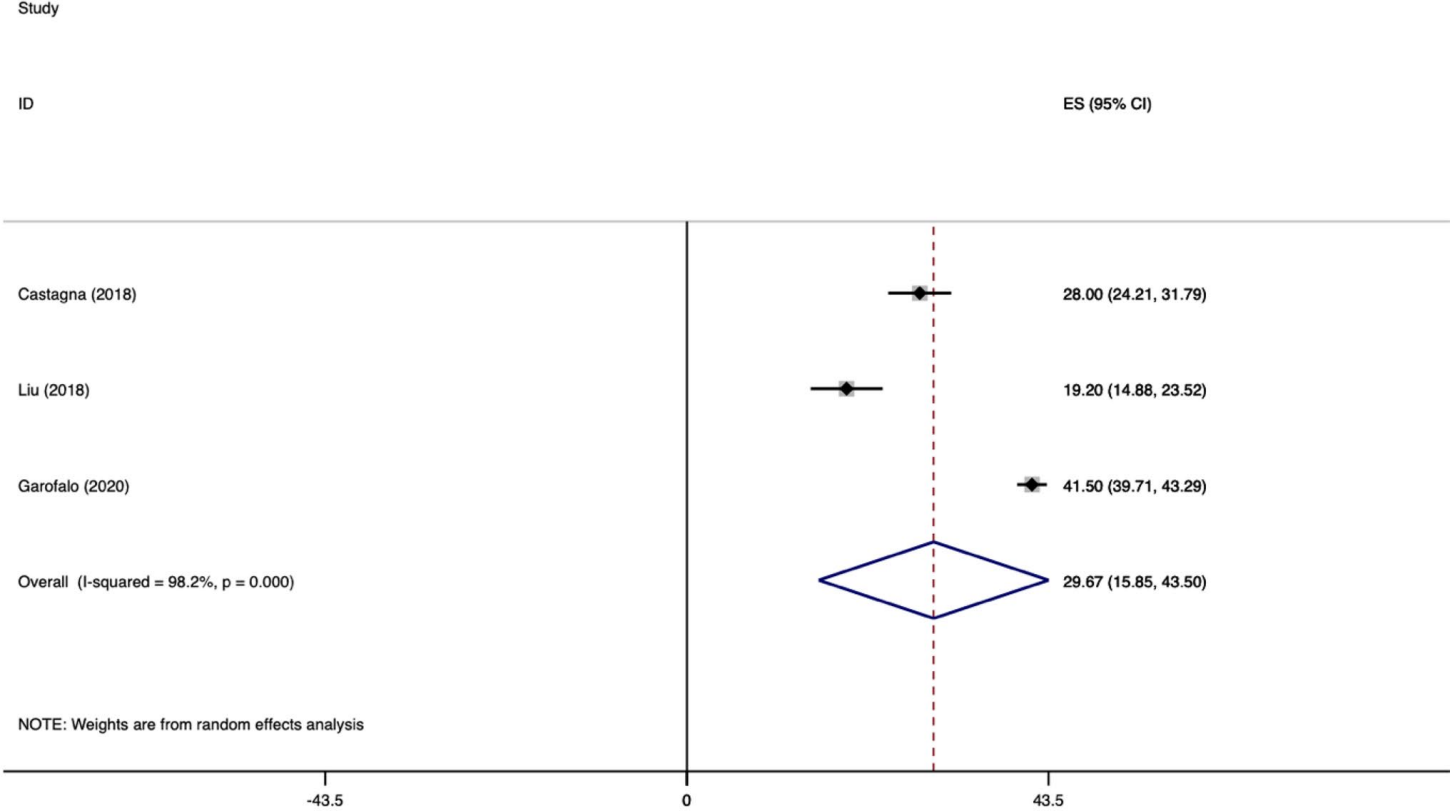
Weighted mean difference of CONSTANT score for TO group

The weighted mean difference of Constant scores for TO (transosseous) group is showed in Fig. 2.

**Fig. 3:**
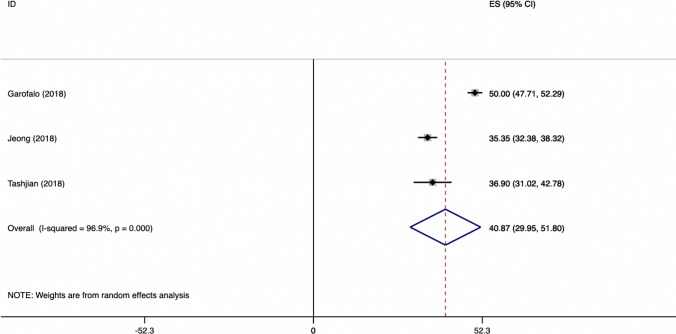
Weighted mean difference of ASES score for SR group

The weighted mean difference of ASES for SA group is showed in Fig. 3.

**Fig. 4:**
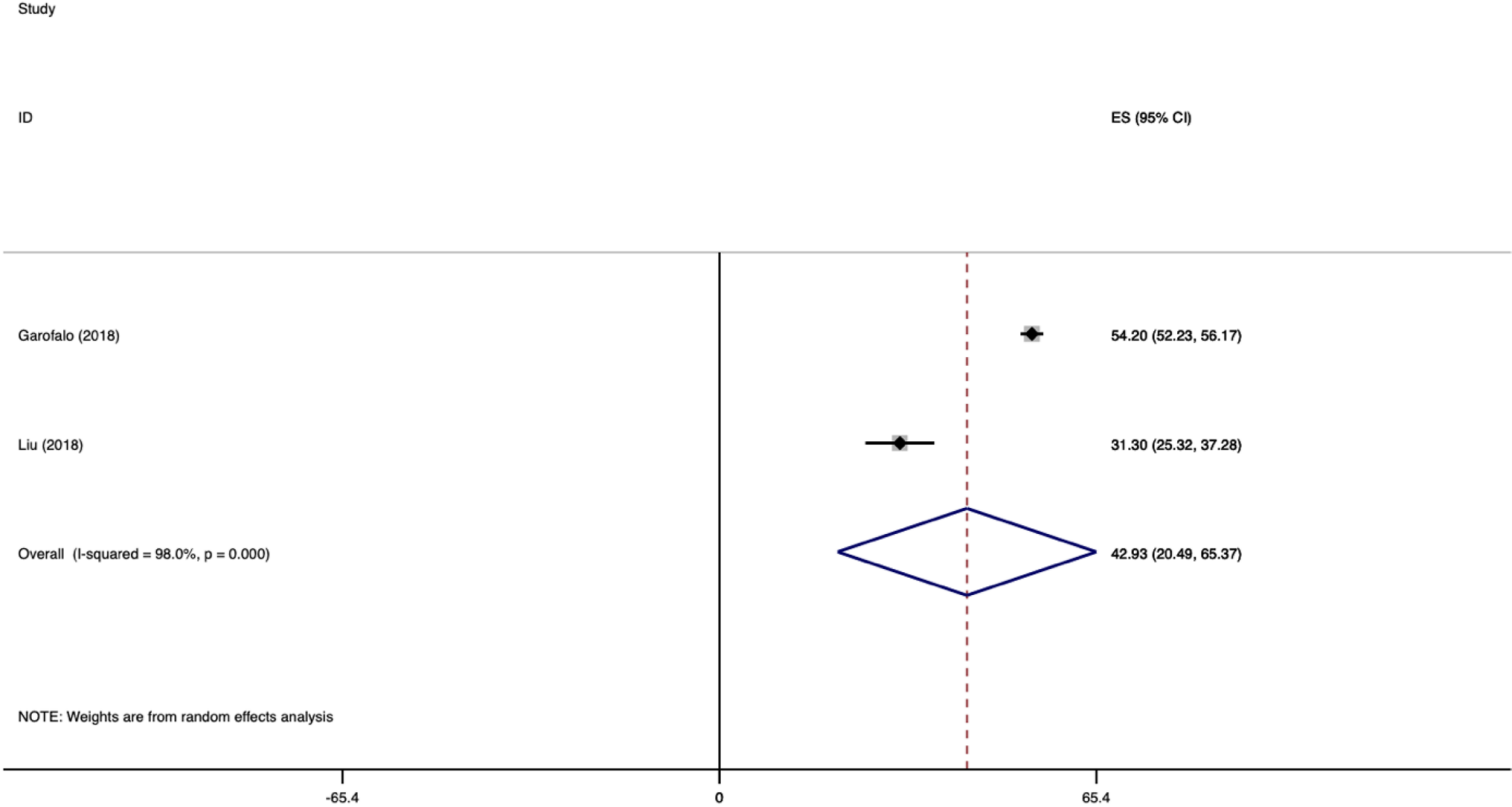
Weighted mean difference of ASES score for TO group

The weighted mean difference of ASES for TO group is showed in Fig. 4.

**Fig. 5:**
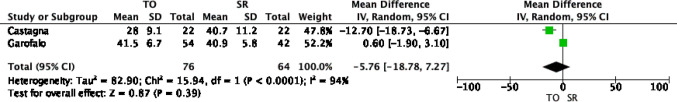
Weighted mean difference of constant for TO versus SR group

The weighted mean difference of CONSTANT for TO versus SA groups is showed in Fig. 5.

No differences were showed in the outcomes of SA and TO techniques for the repair of Rotator Cuff Tears at minimum 24 months follow-up.

## Discussion

The main feature of this Meta-analysis is that there is no difference in the final outcome of the two techniques: SR versus TO technique.

Transosseous rotator cuff tear repair, described by McLaughlin in 1944, has represented the gold standard for years [26]. The advent of arthroscopy has brought a new framework in rotator cuff surgery, supported by the introduction of many devices for fixation over the past few years: screwed or impacted anchors, made of different materials, can be arranged using many different types of repair configurations [22]. SR, DR and TOE anchor-based repair methods are well-described in the literature and have consistently demonstrated good clinical outcomes and healing rates [9]. However shortcomings remain with this technique, such as difficulties with revision surgery, due to the presence of anchors in the greater tuberosity, anchor dislodgement, knot impingement (24) and, eventually, greater tuberosity bone osteolysis [18, 22].

Arthroscopic transosseous technique has been developed to overcome these limitations, combining the minimal invasiveness of the arthroscopic procedures with the biomechanical advantages of the open procedures [11].

The Transosseous Repair is considered more “biological” because of the release of growth factors from bone tunnels which can provide a better healing of the tendon repair [6]. The absence of hardware is another advantage, above all in case of revisions.

The time of surgery is not different between the two techniques [31].

Considering the used scores in the analyzed papers, the main difference between the Constant Score and the ASES Score is that the last one contains also some questions about the daily living activities and the use of pain killers drugs, which are not considered into the Constant Score.

One strength of the study is that it compares the published selected studies with a comparison of single row and Transosseous arthroscopic sutures [6, 12]. Rotator cuff treated with suture-anchors technique were considered for some studies and the technique of transosseous repair was considered for another one [14, 15, 22, 34].

The limitations of the study are that it was not possible to assess potential publication bias because of the few included studies. Furthermore, any sub-analysis for the quality was assessed for the same reason.

## Conclusions

Arthroscopic transosseous rotator cuff repair technique and SA (suture-anchor) technique both lead to significant short-term improvement and satisfactory subjective outcome scores with low complication/failure rates [5, 8, 11]. No differences were found in the final outcome between the two techniques.
